# A Comparison of Short-Term Clinical Outcomes Between the Navitor and Evolut Transcatheter Aortic Valve Prostheses

**DOI:** 10.3390/jcm14165890

**Published:** 2025-08-21

**Authors:** Caterina Campanella, Katherine Kaiser, Stephanie Voss, Melchior Burri, Magdalena Erlebach, Nazan Puluca, Felix Wirth, Markus Krane, Hendrik Ruge

**Affiliations:** 1 Department of Cardiovascular Surgery, Institute Insure, German Heart Center Munich, School of Medicine & Health, Technical University of Munich, 80636 Munich, Germanyvosss@dhm.mhn.de (S.V.);; 2 DZHK (German Centre for Cardiovascular Research)—Partner Site Munich Heart Alliance, 10785 Berlin, Germany; 3 Division of Cardiac Sugery, Department of Surgery, Yale School of Medicine, New Haven, CT 06510, USA

**Keywords:** self-expandable, TAVR, intra-annular, supra-annular, hemodynamic outcomes

## Abstract

**Background:** The novel Navitor intra-annular self-expandable transcatheter aortic valve prosthesis is designed to improve coronary access, reduce paravalvular leaks, and enhance hemodynamic performance. Comparative data with the established Evolut platform (R, Pro, FX) are still lacking. This study aimed to evaluate the short-term clinical outcomes of Navitor (NAV) versus Evolut transcatheter heart valves. **Methods:** We conducted a single-center analysis of patients undergoing transfemoral TAVR between January 2015 and May 2024. A propensity score matching protocol including 18 baseline variables was used to balance baseline characteristics. Clinical outcomes were assessed using VARC-3 criteria. **Results:** Of 1067 TAVR patients, 210 were analyzed after matching—70 with the Nav valve and 140 with the Evolut valve. Baseline characteristics were comparable between groups, with a mean age of 80.9 ± 6.5 years in the NAV group and 80.7 ± 6.7 years in the Evolut group (*p* = 0.9). Both groups had an intermediate STS predicted risk of mortality, 3.9 ± 3.4% for NAV and 3.9 ± 3.2% for Evolut (*p* = 1.0). The effective aortic annulus diameter was comparable between the NAV (23.9 ± 1.5 mm) and Evolut group (23.9 ± 2.4 mm, *p* = 0.8). Hemodynamic performance at discharge was similar, with mean gradients of 7.5 ± 2.8 mmHg (NAV) vs. 7.4 ± 3.7 mmHg (Evolut, *p* = 0.9). Valve orifice areas and paravalvular leak rates showed no difference between the groups. Device success rates at discharge were good for both THVs: 89.3% for Evolut and 91.4% for NAV (*p* = 0.8). Disabling stroke occurred less frequently in the NAV group (0.0% vs. 7.1%, *p* = 0.033), while other early safety outcomes and 30-day mortalities were similar. **Conclusions:** The Navitor valve offers comparable hemodynamic performance, paravalvular leak rates, and procedural success to the Evolut platform. While early safety outcomes were largely similar, the Navitor valve was associated with a lower 30-day disabling stroke rate.

## 1. Introduction

Transcatheter aortic valve replacement (TAVR) has revolutionized the treatment of patients with severe aortic stenosis, particularly those at high or intermediate surgical risk [1,2]. Over the past decade, the availability of multiple transcatheter heart valve (THV) platforms and continuous device improvements has led to significant enhancements in TAVR clinical outcomes. One of the most frequently used devices is the Evolut (Medtronic, Minneapolis, MN, USA) system, featuring a supra-annular, self-expanding design [3]. The recently FDA-approved and CE-certified Navitor (Abbott, Abbott Park, IL, USA) prosthesis shares key characteristics with the Evolut system, including a self-expanding release mode, the option of valve recapturability, and a deployment mechanism without the need of rapid pacing. However, a significant difference between the two types of prosthesis is the implantation height in relation to the native annulus [3,4,5]. The Navitor prosthesis utilizes an intra-annular design, where the leaflets are positioned at the level of the aortic annulus [5]. This distinction may have important implications for clinical outcomes. The valve expansion in the intra-annular position might be restricted by the surrounding annulus, potentially leading to a smaller orifice area. In contrast, supra-annular valves have been shown to provide a larger orifice area, which may enhance hemodynamic performance [1,2,6]. Furthermore, the intra-annular positioning may offer better access to the coronary arteries for future diagnostic or interventional procedures [3,4,5]. This is an increasingly important consideration as younger patients with longer life expectancies undergo TAVR and may later develop coronary artery disease. Comparative data analyzing the clinical outcomes between these two valve designs are currently lacking. The aim of this study is to compare short-term clinical outcomes following TAVR with the intra-annular self-expandable Navitor (NAV) valve and the supra-annular self-expandable Evolut (R, Pro, FX) transcatheter heart valves (THVs).

## 2. Materials and Methods

### 2.1. Study Design and Patient Selection

All patients who underwent transfemoral TAVR with either a Navitor (NAV) or Evolut (R, Pro, FX) transcatheter heart valve (THV) between January 2015 and May 2024 were identified from our institutional database. Patients with non-transfemoral access, valve-in-valve procedures, bicuspid aortic valve anatomy, or missing data were excluded. Patients with missing variables necessary for propensity score matching were excluded from the study. Propensity score matching (PSM) was performed using 18 variables to balance baseline characteristics. Optimal balance was achieved with a 1:2 matching ratio, resulting in 70 Navitor patients matched to 140 Evolut patients.

Baseline demographics, clinical and echocardiographic data, preprocedural CT findings, procedural details, and follow-up outcomes were prospectively recorded by coinvestigators and stored in the institutional TAVR database. MSCT Ecg guided contrast enhanced 1 mm slice thickness protocol was applied. Imaging data sets were analyzed using 3mensio Structural Heart (v10.4; Pie Medical Imaging, Maastrich, The Netherlands) software for automated three-dimensional CT reconstruction. 

Study assessments were conducted at baseline, discharge, 30 days, and 6 months. Clinical follow-up was completed for all patients up to 6 months post-TAVR. Missing data were retrieved via phone follow-up or discharge summaries from referring physicians. Clinical outcomes were defined according to Valve Academic Research Consortium-3 (VARC-3) [7] criteria, including device success at discharge, all-cause mortality, disabling stroke, life-threatening bleeding, and major vascular complications within 30 days. Additional endpoints included new pacemaker implantation, transvalvular gradients, and paravalvular regurgitation. Per VARC-3 guidelines, any death of unknown cause was classified as cardiovascular mortality.

### 2.2. Transcathter Heart Valve Systems

#### 2.2.1. The Navitor (Abbott, Abbott Park, IL, USA)

**Figure 1:**
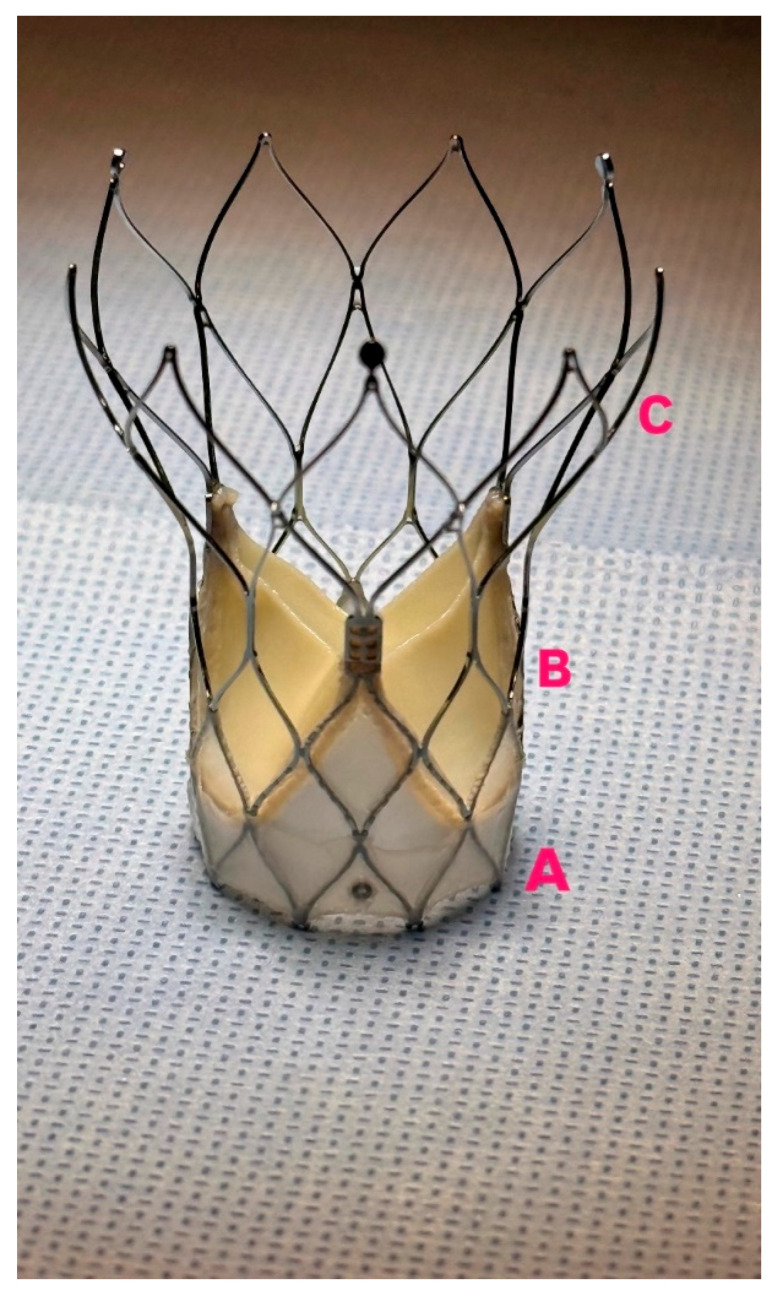
Navitor Prothesis, (**A**): NaviSeal cuff, (**B**): Intra-annular leaflets positioning, (**C**): large stent cells.

The Navitor THV features a heat-sensitive titanium–nickel alloy frame with a large cell design, bovine pericardial leaflets, and an intra-annular leaflet configuration. It includes the Naviseal™ polyethylene sealing cuff for PVL mitigation and improved radial force distribution through enhanced materials engineering. The FlexNav™ delivery system offers a hydrophilic-coated integrated sheath for controlled deployment and reduced minimum vessel access sizes (5 mm for smaller and 5.5 mm for larger valves) [4] (Figure 1).

#### 2.2.2. Evolut (Medtronic, Minneapolis, MN, USA)

**Figure 2:**
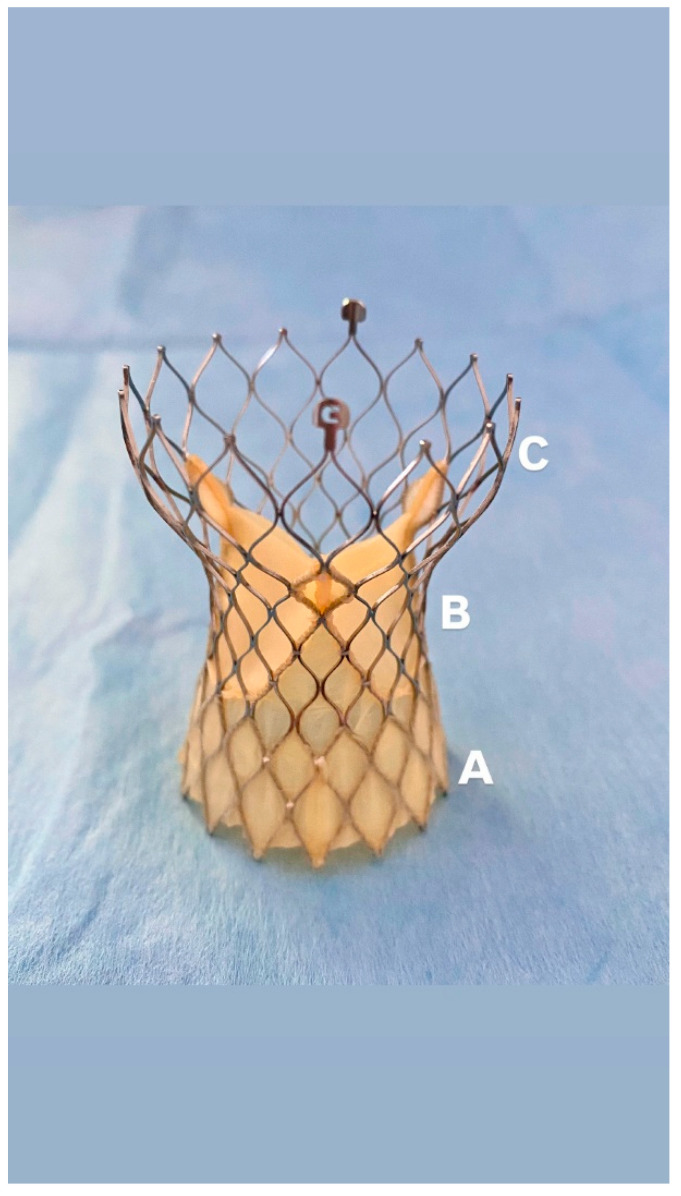
Evolut FX Prosthesis: (**A**): Porcine pericardial wrap, (**B**): Supra-annular leaflets positioning, (**C**): self-expanding nitinol frame.

The Evolut™ valve platform is a self-expanding transcatheter heart valve system composed of a nitinol frame and porcine pericardial leaflets positioned in a supra-annular location. It is recapturable and repositionable during deployment. Two of the latest designs in the platform are the Evolut™ PRO and Evolut™ FX. The Evolut PRO includes an external porcine pericardial wrap covering the lower 1.5 cells (approximately 12 mm) to improve annular sealing and reduce paravalvular leak. The Evolut FX retains the same valve structure but incorporates modifications to the delivery system for increased flexibility and trackability [8] (Figure 2).

### 2.3. Statistical Analysis

Descriptive statistics were used to summarize baseline characteristics and propensity score matching (1:2) was performed to adjust for baseline differences between the two groups. The matching variables included age, gender, STS predicted risk of mortality, Euroscore II, BMI, aortic annular parameters (diameter, perimeter), aortic valve parameters (preoperative AV mean pressure gradient, preoperative ≥ moderate aortic insufficiency), ≥moderate mitral regurgitation, left ventricular function, atrial fibrillation, preoperative pacemaker, peripheral arterial disease, carotid artery disease, previous history of stroke, COPD and creatinine levels. Continuous variables were compared using t-tests, and categorical variables were compared using chi-square tests or Fisher’s exact tests where appropriate. Statistical significance was defined as a *p*-value < 0.05 and all statistical analyses were performed using R. For matching, a non-parsimonious multivariate logistic regression-model was calculated using the baseline variables. The model was used to calculate the propensity score in each patient. Then, propensity score matching was performed with the ratio of 1:2. The standardized mean difference was calculated to evaluate difference in each baseline characteristics before and after matching.

## 3. Results

### 3.1. Patients

**Figure 3:**
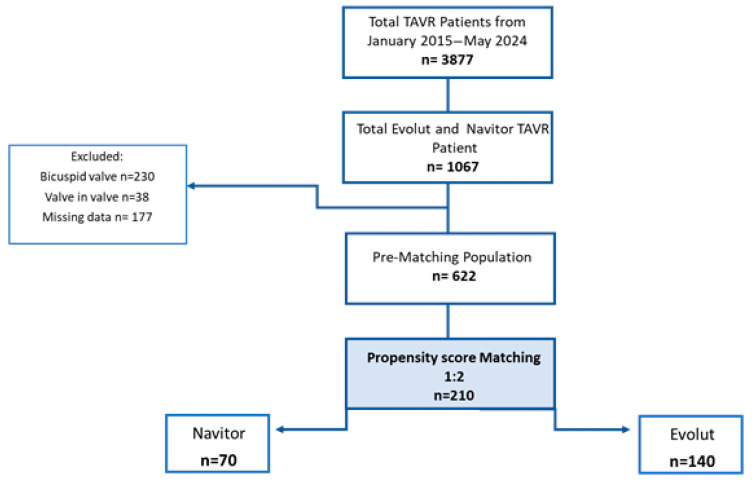
Flow diagram of study.

Between January 2015 and May 2024, a total of 3877 patients underwent a TAVR procedure at the Department of Cardiovascular Surgery at the German Heart Center Munich. Among them, we identified 1067 patients treated with either the Evolut or NAV prosthesis via transfemoral access. In total, 445 patients were excluded from the study analysis due to a bicuspid aortic valve *(n* = 230), valve in valve procedures (*n* = 38), and missing data sets (*n* = 177). After 1:2 propensity score matching, the final cohort resulted in 210 patients: 70 NAV patients vs. 140 Evolut patients (Figure 3).

### 3.2. Baseline Characteristics

Prior to matching, there was a significant difference in Euroscore II between the Navitor and Evolut groups. The Navitor group had a higher operative risk, with an average Euroscore II of 3.3 ± 2.9 compared to 4.9 ± 5.2 in the Evolut group (*p* = 0.001). Additionally, there was a notable difference in annulus perimeter, with the Navitor group having a larger anatomy, showing an annulus perimeter of 7.6 ± 0.5 mm compared to 7.4 ± 0.7 mm in the Evolut group (*p* = 0.01). After applying the matching protocol, the two cohorts were comparable regarding operative risk and annular anatomy, and, overall, were well balanced. However, characteristics such as COPD, female gender, and carotid artery disease remained unbalanced between the groups.

After matching, the mean age for NAV and Evolut patients was 80.9 ± 6.5 years vs. 80.7 ± 6.7 years (*p* = 0.9), with 45.7% females in the NAV group and 51.4% females in the Evolut group (*p* = 0.5). Both cohorts exhibited an intermediate Society of Thoracic Surgery predicted risk of mortality of 3.9 ± 3.4% (NAV) and 3.9 ± 3.2% (Evolut) (*p* = 1.0). Further baseline characteristics, as well as preoperative echocardiographic data, are displayed in Table 1.

### 3.3. Procedural Outcomes and VARC 3 Defined Technical and Device Success Rates

VARC-3 technical success at the end of the procedure was comparable for both THVs (Navitor 95.7% vs. Evolut 94.3%, *p* = 0.8). Technical failure was primarily due to valve embolization in the NAV group, with three patients (4.3%) requiring surgical aortic valve replacement. In the Evolut group, one patient was converted to surgical aortic valve replacement due to valve embolization, and seven patients (5%) received a second valve due to severe paravalvular insufficiency. The Nav group required a significantly higher volume of contrast medium compared to the Evolut group (144 [115–200] mL vs. 120 [95–142] mL, *p* < 0.001), while procedure duration was similar between the two groups (62 [54–69] min vs. 60 [50–71] min, *p* = 0.7). Further details on procedural outcomes as well as on the number and size of the prostheses used can be found in Table S1 of the Supplementary Materials (Supplement Table S1: Procedural data).

VARC-3 device success at discharge was similar between the two THV platforms (Nav 95.7% vs. Evolut 94.3%, *p* = 0.8). The reasons for device failure are listed in the Supplementary Materials (Supplement Table S2: Clinical outcomes: Technical and Device failure reason).

### 3.4. Hemodynamic Outcomes

**Figure 4:**
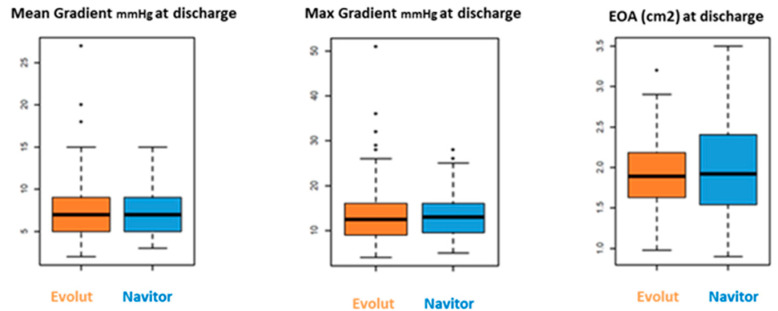
Hemodynamics outcomes.

Hemodynamic outcome at discharge showed similar results for both cohorts: mean gradient for the NAV group was 7.5 ± 2.8 mmHg vs. 7.4 ± 3.7 mmHg for the Evolut group (*p* = 0.9), and maximum gradient was 13.6 ± 5.3 mmHg for NAV vs. 13.7 ± 6.9 mmHg for Evolut (*p* = 0.9). Aortic valve orifice area was similar in both groups (NAV 2.0 ± 0.7 cm^2^ vs. Evolut 1.9 ± 0.6 cm^2^, *p* = 0.3) (Figure 4).

Most patients had none/trace or mild paravalvular regurgitation (PVL): 42.9% (NAV) vs. 45.7% (Evolut) had none/trace (*p* = 0.6), and 50.0% (NAV) vs. 48.6% (Evolut) had mild regurgitation (*p* = 0.6) (Table 2).

### 3.5. Clinical Outcomes

The rate of disabling stroke at 30 days was significantly lower in the NAV group (0.0% vs. Evolut 7.1%, *p* = 0.033). The need for permanent pacemaker implantation (PPI) was higher in the Evolut group without statistical significance (12.9% vs. 15.7%, *p* = 0.7). A subanalysis of the post-Evolut PPI rate before and after 2020 showed a lower PPI rate after 2020 compared to prior years (6.8% after 2020 vs. 19.8% before 2020, *p* = 0.11). Vascular complications, including both minor and major events, were comparable between the two groups (minor: 17.1% for NAV vs. 12.9% for Evolut; major: 2.9% for NAV vs. 7.9% for Evolut, *p* = 0.8). Major bleeding adverse events occurred more frequently in the Evolut group, without reaching statistical significance (Nav 1.4% vs. Evolut 5.7%, *p* = 0.08). Further details of perioperative clinical outcomes are displayed in Table 3.

### 3.6. 30 Days and 6 Months—Survival

**Figure 5:**
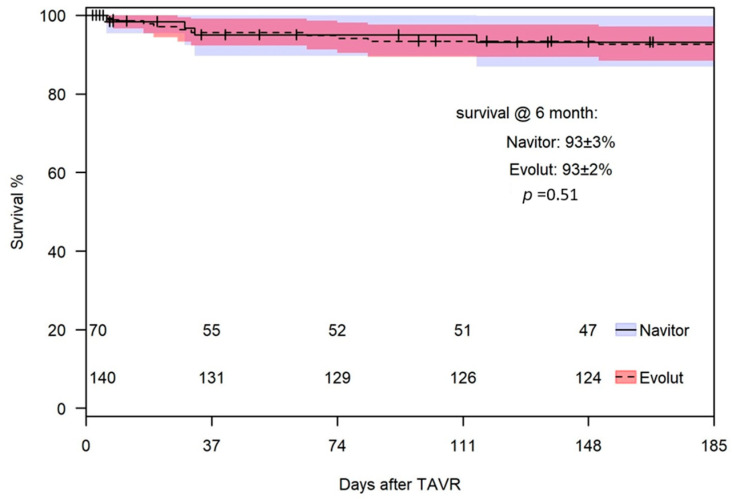
Survival at 6 months.

The 30-day all-cause mortality rate was similar for both THVs, with two deaths (2.9%) in the NAV group and five deaths (3.6%) in the Evolut group (*p* = 1.0). Four patients died from cardiovascular causes (one with NAV and three with Evolut), while three died from non-cardiovascular causes (one with NAV and two with Evolut). The survival rate at 6 months was 92 ± 4% for the NAV group and 93 ± 2% for the Evolut group (*p* = 0.51) (Figure 5).

At 6 months, echocardiographic data were available for 53 (80.3%) of 66 eligible patients in the NAV group and 108 (83.1%) of 130 eligible patients in the Evolut group. Moderate PVL was observed in two (3.7%) NAV THVs and one (0.9%) Evolut THV (*p* = 0.2). A transvalvular gradient >20 mmHg was found in two (1.9%) Evolut patients and none in the NAV group (*p* = 1.0).

## 4. Discussion

This study provides, to our knowledge, the first comparison of short-term clinical outcomes following TAVR with the Navitor versus Evolut THVs in a real-world population. The main results of the study are as follows:

The intra-annular Navitor valve showed similar single-digit mean transvalvular gradients, effective orifice area, and paravalvular leak rates compared to the supra-annular Evolut platform.

VARC-3 device success rates at discharge were high for both THV platforms with low rates of adverse events up to 30 days after TAVR.

All-cause mortality was comparable between the two groups at 30 days and six months. At six months, no significant differences were observed in the incidence of in ≥moderate PVL and in transvalvular gradients exceeding 20 mmHg.

### 4.1. Hemodynamic Outcomes

In our study, both the Evolut and NAV transcatheter heart valves (THVs) demonstrated promising results in terms of PVL, with only one patient in the NAV group showing moderate PVL at discharge. These findings are consistent with the Portico NG trial, which reported no moderate or severe PVL after NAV implantation in high- or extreme-risk surgical patients at 30 days [4]. Similarly, our results align with the Optimize Pro trial, where 78% of Evolut (PRO/PRO+) patients had none or trace PVL, with no instances of moderate or high grade PVL at 30 days [9]. These data suggest that THV platforms achieve comparable PVL outcomes. In terms of hemodynamic performance, both the Evolut and Navitor valves demonstrated favorable outcomes, with single-digit mean transvalvular gradients following TAVR. The mean gradient in the Evolut group (7.3 ± 3.8 mm Hg) was consistent with previously reported values from studies of the Evolut PRO+ and FX generations [10,11]. Similarly, the Navitor group exhibited mean gradients in line with those observed in the Portico NG trial [4]. One possible explanation for the low mean gradient observed with the Navitor valve, despite its intra-annular leaflet position, is its unique cylindrical inflow design [4]. This geometry may promote more complete leaflet opening compared to the tapered inflow of the Evolut stent frame, which may impose a mild constraint on leaflet motion. A similar limitation has been observed with the Sapien valve, which also features a tapered leaflet design and has shown slightly higher transvalvular gradients [12]. As expected, the Evolut valve, with its supra-annular leaflet position, was associated with a large effective orifice area (2.0 ± 0.5 cm^2^), consistent with the prior literature [9,11,13,14]. Notably, the Navitor valve achieved a comparable orifice area (1.9 ± 0.4 cm^2^), despite its intra-annular design, supporting the growing body of evidence that design optimizations in newer intra-annular valves can yield hemodynamic performance like that of supra-annular platforms [4].

### 4.2. Device Success Rate and Clinical Outcomes

Device success rates at discharge were high for both THVs: 89.3% for Evolut and 91.4% for NAV (*p* = 0.8). Regarding clinical outcomes, the NAV group had 2.9% major vascular complications, compared to 7.9% in the Evolut group, though this difference was not significant (*p* = 0.8). The lower rate of vascular complications in the NAV group may be due to the FLEX NAV sheath, designed for better vascular access in challenging anatomies, matching the Portico NG trial’s vascular complication rate of 0.8% [3,4]. The higher complication rate in the Evolut group may reflect earlier model performance, as sheath modifications were made over time to improve access [15]. Permanent pacemaker implantation (PPI) rates were comparable between the NAV group (12.9%) and the Evolut group (15.7%) (*p* = 0.7), and were in line with reported rates for other TAVR devices, including the Evolut PRO (11.8%), ACURATE neo2 (15.0%), and SAPIEN 3 (13.3%) [13,16,17]. A subgroup analysis of PPI rates in the Evolut cohort demonstrated a trend toward reduced pacemaker implantation following the adoption of the cusp overlap technique (COT) after 2020 (6.8% after 2020 vs. 19.8% before 2020, *p* = 0.11) [18]. At 30 days, the rate of disabling stroke was significantly lower following Navitor THV implantation compared to the Evolut platform (0.0% vs. 7.1%, *p* = 0.033). This stroke rate in the Evolut group is higher than those reported in previous large-scale trials, such as Evolut Low-Risk and SURTAVI, which observed rates around 0.5% [1,6]. This difference should be interpreted with caution, as the event rate was too low to analyze the underlying reasons. These findings have generated hypotheses for future studies. Notably, stroke events in the Evolut cohort were not evenly distributed over time. Of the 10 total disabling strokes, 7 (70%) occurred between 2015 and 2019, while the remaining 3 strokes (30%) occurred between 2020 and 2024. This may suggest a trend toward decreasing stroke incidence in later years, possibly reflecting improved procedural techniques, increasing operator experience, and ongoing device evolution. While these observations suggest a potentially favorable neurologic safety profile for the Navitor valve, further investigation in larger, randomized studies is warranted to confirm these findings

### 4.3. Follow-Up and Mortality Rate

The NAV THV features the Naviseal cuff, which has been shown to result in over 70% of patients having none or trace PVL up to 1 year post-procedure [3,4,5]. At 6 months, only two (3.7%) NAV valves and one (0.9%) Evolut valve had moderate PVL (*p* = 0.2), indicating comparable performance and low rates of moderate regurgitation. The NAV prosthesis also showed excellent transvalvular gradients, with no patients exceeding a gradient of 20 mmHg at 6 months, compared to two patients in the Evolut group. The Portico NG trial reported a slightly better 1-year PVL rate (1% vs. 3.7% in our cohort); however, our findings align with those of contemporary TAVR devices, which report PVL rates ranging from 0.6% to 2.7% [16,17,19,20].

The 30-day all-cause mortality rate was similar for both THV systems, with two (2.9%) deaths in the NAV group and five (3.6%) in the Evolut group (*p* = 1.0). Other cohort studies reported 30-day mortality rates ranging from 0% to 2.4% [4,17], slightly lower than in our study, which could be attributed to the broader time frame of our analysis, which started in 2015. Notably, some patients in both groups died due to non-cardiovascular reasons not related to the type of THVs.

### 4.4. Future Perspective

While long-term data on the well-established Evolut platform are readily available in the literature, evidence regarding the future durability and hemodynamic performance of the novel Navitor valve remains limited. Our study provides only 6-month follow-up data; however, extended follow-up beyond 1 year is essential to better evaluate the long-term performance and durability of the Navitor THV. Coronary access remains a key consideration, particularly given the differences in stent frame design between the two THVs. The Evolut platform benefits from manufacturer-guided deployment instructions that aid in commissure alignment, improving coronary access. In contrast, the NAV THV, with its larger stent frame cells, may offer advantages for catheter navigation in coronary access. However, it lacks specific deployment recommendations, which may pose challenges for optimal commissure alignment. Further studies are needed to better evaluate how the NAV deployment affects coronary access.

### 4.5. Limitations

This study has several limitations. First, its retrospective design and relatively short-to-midterm follow-up limit the ability to make definitive conclusions, particularly regarding long-term durability. Specifically, the 6-month follow-up period is insufficient to assess the long-term performance of the Navitor valve. Additionally, the calcium load of the native valve was not assessed, which may influence stroke rates. While propensity score matching was employed to reduce selection bias, unmeasured variables may still affect the results. Finally, given the technological advancements in the Evolut platform over time, future studies should compare newer generations of both devices to mitigate potential bias arising from differences in deployment periods.

## 5. Conclusions

The novel intra-annular self-expandable Navitor valve demonstrates similar single-digit mean transvalvular gradients, similar effective orifice areas, and similar paravalvular leak rates when compared to the supra-annular Evolut platform. Device success rates were high for both valve types. While early safety outcomes were largely similar, the Navitor valve was associated with a lower 30-day disabling stroke rate.

## Figures and Tables

**Table 1:** Patients’ characteristics and baseline clinical data.

	Pre-Matched				Post-Matched			
	Navitor n = 70	Evolut n = 552	SMD	*p* -val	Navitor n = 70	Evolut n = 140	SMD	*p* -val
**Age (y)**	80.9 ± 6.5	81.0 ± 6.2	0.019	0.9	80.9 ± 6.5	80.7 ± 6.7	0.024	0.9
**STS-PROM**	3.9 ± 3.4	4.1 ± 3.6	0.061	0.6	3.9 ± 3.4	3.9 ± 3.2	0.005	1.0
**EuroSCORE II**	3.3 ± 2.9	4.9 ± 5.2	0.322	<0.001	3.3 ± 2.9	3.4 ± 2.9	0.037	0.8
**BMI (kg/m^2^)**	26.9 ± 4.3	27.3 ± 5.1	0.065	0.6	26.9 ± 4.3	26.5 ± 5.0	0.091	0.5
**Female gender (%)**	32 (45.7%)	312 (56.5%)	0.217	0.1	32 (45.7%)	72 (51.4%)	0.114	0.5
**PAD (%)**	7 (10.0%)	85 (15.4%)	0.152	0.3	7 (10.0%)	7 (5.0%)	0.200	0.2
**Prev. stroke (%)**	8 (11.4%)	68 (12.3%)	0.027	1.0	8 (11.4%)	18 (12.9%)	0.043	0.8
**Carotid art. dis. (%)**	6 (8.6%)	54 (9.8%)	0.042	1.0	6 (8.6%)	8 (5.7%)	0.114	0.6
**CAD (%)**	44 (62.9%)	306 (55.4%)	0.150	0.3	44 (62.9%)	90 (64.3%)	0.030	0.9
**Prev. pacemaker (%)**	4 (5.7%)	52 (9.4%)	0.129	0.4	4 (5.7%)	10 (7.1%)	0.057	0.8
**Atrial fibrillation**	24 (34.3%)	164 (29.7%)	0.100	0.5	24 (34.3%)	46 (32.9%)	0.030	0.9
**COPD (%)**	12 (17.1%)	58 (10.5%)	0.210	0.1	12 (17.1%)	17 (12.1%)	0.145	0.4
**Serum creatinin (mg/dL)**	1.1 ± 0.5	1.2 ± 0.8	0.203	0.03	1.1 ± 0.5	1.1 ± 0.4	0.060	0.7
**LVEF %**	56.7 ± 11.0	54.3 ± 11.8	0.202	0.1	56.7 ± 11.0	56.5 ± 11.1	0.021	0.9
**AV mean gradient (mmHg)**	42.5 ± 16.6	39.5 ± 15.4	0.196	0.1	42.5 ± 16.6	40.9 ± 16.8	0.096	0.5
**Aortic regurg ≥ mod.**	4 (5.7%)	41 (7.4%)	0.070	0.8	4 (5.7%)	8 (5.7%)	0.005	1.0
**Mitral regurg ≥ mod.**	6 (8.6%)	62 (11.2%)	0.085	0.7	6 (8.6%)	14 (10.0%)	0.049	0.8
**Annulus perimeter (cm)**	7.6 ± 0.5	7.4 ± 0.7	0.258	0.01	7.6 ± 0.5	7.6 ± 0.8	0.016	0.9
**Annulus diameter (mm)**	23.9 ± 1.5	23.5 ± 2.3	0.166	0.07	23.9 ± 1.5	23.9 ± 2.4	0.024	0.8

**Table 2:** Hemodynamics clinical outcomes at discharge.

	Navitor n = 70	Evolut n = 140	*p* -Value
**AV PG max mmhg**	13.6 ± 5.3	13.7 ± 6.9	0.9
**AV PG mean mmhg**	7.5 ± 2.8	7.4 ± 3.7	0.9
AV EOA cm2	2.0 ± 0.7	1.9 ± 0.6	0.3
**AV regurg**			0.6
--none	30 (42.9%)	64 (45.7%)	
--mild	35 (50.0%)	68 (48.6%)	
--mild–moderate	4 (5.7%)	8 (5.7%)	
--moderate	1 (1.4%)	0 (0.0%)	

**Table 3:** Clinical outcomes.

	Navitor N = 70	Evolut N = 140	*p* -Value
Intraprocedural death (%)	0 (0.0%)	0 (0.0%)	
Technical success	67 (95.7%)	132 (94.3%)	0.8
Device success at discharge	64 (91.4%)	125 (89.3%)	0.8
All stroke	0 (0.0%)	10 (7.1%)	0.033
Bleeding			0.08
--minor	1 (1.4%)	9 (6.4%)	
--2_major	1 (1.4%)	8 (5.7%)	
--3_lifethreatening/disabling	2 (2.9%)	3 (2.1%)	
Vascular complication			0.8
--1_minor	12 (17.1%)	18 (12.9%)	
--2_major	2 (2.9%)	11 (7.9%)	
Postop PM	9 (12.9%)	22 (15.7%)	0.7
30-days mortality (%)	2 (2.9%)	5 (3.6%)	1.0

## Data Availability

The data that support the findings of this study are available from the corresponding author upon reasonable request.

## Supplementary Materials

Details on procedural outcomes as well as on the number and size of the prostheses used can be found in Table S1 of the Supplementary Materials https://www.mdpi.com/article/10.3390/jcm14165890/s1. Reasons for technical and device failure are listed in Table S2 of the Supplementary Materials.

## Abbreviations

AF	Atrial Fibrillation
AR	Aortic Valve Regurgitation
AV	Aortic Valve
BMI	Body Mass Index
COPD	Chronic Obstructive Pulmonary Disease
LVEF	Left Ventricular Ejection Fraction
NAV	Navitor
MSCT	Multislice Computed Tomography
VARC III	Valve Academic Research Consortium III
PG	Pressure Gradient
PPI	Permanent Pacemaker Implantation
PVL	Paravalvular Leackage
TAVR	Transcatheter Aortic Valve Replacement
THV	Transcatheter Heart Valve
