# Supplementary material for: A Comparison of Short-Term Clinical Outcomes Between the Navitor and Evolut Transcatheter Aortic Valve Prostheses
Source: J Clin Med. 2025 Aug 21;14(16):5890. doi: 10.3390/jcm14165890 (PMC12387559; doi:10.3390/jcm14165890)
Supplement: Supplementary file 1 [file jcm-14-05890-s001.zip › jcm-3787910-supplementary.pdf]

SUPPLEMENT TABLES

Supplement Table S1: Procedural data

	Navitor N=70	Evolut N= 140	P.Value
Intubation (%)	4 (5.7%)	42 (30.0%)	<0.001
Pre-dilatation (%)	69 (98.6%)	57 (40.7%)	<0.001
Post-dilatation (%)	29 (41.4%)	62 (44.3%)	0.8
Pericardial tamponade (%)	1 (1.4%)	1 (0.7%)	1.0
Conversion to surgery (%)	3 (4.3%)	1 (0.7%)	0.11
2nd Valve implanted (%)	0 (0.0%)	7 (5.0%)	0.10
Valve (%)			<0.001
--Navitor 23	2 (2.9%)	0 (0.0%)	
--Navitor 25	17 (24.3%)	0 (0.0%)	
--Navitor 27	30 (42.9%)	0 (0.0%)	
--Navitor 29	21 (30.0%)	0 (0.0%)	
--Evolut (R, FX, Pro, Pro+) 23	0 (0.0%)	4 (2.9%)	
--Evolut (R, FX, Pro, Pro+) 26	0 (0.0%)	47 (33.6%)	
--Evolut (R, FX, Pro, Pro+) 29	0 (0.0%)	62 (44.3%)	
--Evolut (R, FX, Pro, Pro+) 34	0 (0.0%)	27 (19.3%)	

Supplement Table S2: Clinical outcomes: Technical and Device failure reason

Label	Navitor<br>n=70	Evolut<br>n=140	P-Value
Technical success at exit from hybrid OR (%)	67 (95.7%)	132 (94.3%)	0.8
Reason for not reaching technical success (%)			
-- 2nd valve used	0 (0.0%)	7 (5.0%)	0.10
-- conversion to surgery	3 (4.3%)	1 (0.7%)	0.11
-- malplacement	0 (0.0%)	1 (0.7%)	1.0
Device Success at discharge (%)	65 (92.9%)	126 (90.0%)	0.8
Reason for not reaching device success (%)			
-- gradient (mean gradient > 20mmHg)	0 (0.0%)	2 (1,42%)	0.3
-- intrahospital death	1 (2.9%)	4 (3.6%)	1.0
-- PVL (≥ moderate)	1 (1.4%)	0 (0.0%)	0.3
